# Beyond Resistance Genes: Silencing Susceptibility

**DOI:** 10.3390/ijms27072938

**Published:** 2026-03-24

**Authors:** Anuradha De Silva, Wannakuwattewaduge Gerard Dilantha Fernando

**Affiliations:** 1 Department of Plant Science, University of Manitoba, Winnipeg, MB R3T 2N2, Canada; j.desilva@umanitoba.ca; 2 St. Paul’s College, University of Manitoba, Winnipeg, MB R3T 2M6, Canada

**Keywords:** resistance genes, susceptibility genes, broad-spectrum resistance, OMICS tools

## Abstract

The ongoing evolutionary struggle between crops and pathogens has highlighted the limitations of resistance gene (*R* gene)-based control, which often fails due to rapid pathogen adaptation. To address this, we must look beyond R genes and explore susceptibility genes (S genes) that pathogens take over during infection. Recent success stories suggest that S-gene manipulation can provide broad-spectrum resistance. However, moving this technology to the field requires careful balancing to ensure that plant fitness is not compromised. In this review, we focus on integrated multi-omics tools to identify new avenues for resistance, specifically the mechanisms of R-gene breakdown and the potential of omics-driven strategies for long-term crop protection.

## 1. Introduction

Plant immunity is traditionally framed by the Zig-Zag Model, consisting of two interconnected systems: pattern-triggered immunity (PTI) and effector-triggered immunity (ETI) [1,2]. PTI serves as the broad-spectrum first line of defense, where surface-localized pattern recognition receptors (PRRs) detect Pathogen-Associated Patterns (PAMPs), such as fungal chitin and bacterial flagellin, to trigger baseline defenses like reactive oxygen species (ROS) bursts and cell wall strengthening [3]. To bypass this, pathogens secrete effectors to disable PTI, prompting the evolution of ETI. In this second layer, intracellular nucleotide-binding leucine-rich repeat receptors (NLRs) detect specific effectors. The classic “gene for gene” resistance often results in a hypersensitive response (HR) to arrest pathogen spread [4]. Modern understanding, however, treats PTI and ETI not as isolated layers but as a unified network; major genes (NLRs) often require functional PTI signaling from minor PRR genes to reach their full defensive potential [5,6,7,8]. Conversely, minor genes provide quantitative, broad-spectrum surveillance that maintains cellular machinery, such as MAPK cascades, effectively lowering the threshold for major genes to trigger a robust immune response [9,10,11].

Despite this complex immune network, pathogens have evolved sophisticated “effector suites” to suppress both layers of defense. By deploying effectors that target MAP kinase pathways or even other ETI-triggering effectors, pathogens can effectively hide from host surveillance [12]. The high mutation rates and redundancy within these effector suites allow pathogens to rapidly bypass host genes, leading to the ecologically and economically damaging “boom-and-bust “cycles [13]. Also, pathogen genomes often contain multiple proteins to perform the same task (i.e., several different effectors targeting the same host immune protein), and this redundancy ensures that if the plant evolves to recognize one effector, the pathogen can lose it without losing the ability to infect [14,15]. Moreover, the outcome of an interaction is not determined solely by the plant immune receptors (NLRs and PRRs). It also depends on the presence of host susceptibility (S) genes. These are native plant genes that the pathogen chooses to facilitate infection, provide nutrients, or suppress defense signaling [16]. Unlike *R*-gene-mediated resistance, which is bypassed by evolving pathogen effectors, susceptibility represents a fundamental requirement for the pathogen life cycle within the host.

Most plant breeding focuses on adding *R* genes; however, this often leads to the above-mentioned “boom-and-bust” cycles. Scientists have now discovered a novel way to achieve more durable, broad-spectrum resistance by identifying and disabling these S genes. By modifying or knocking out these susceptibility factors, the plant becomes an inhospitable environment for the pathogen, effectively preventing the suppression of the immune system without relying on race-specific recognition [17,18]. The central theme of this review is how we can identify the breakdown of resistance genes and develop long-lasting immunity using OMICS approaches.

## 2. Mechanisms of R-Gene Breakdown and Pathogen Adaptation

**Figure 1:**
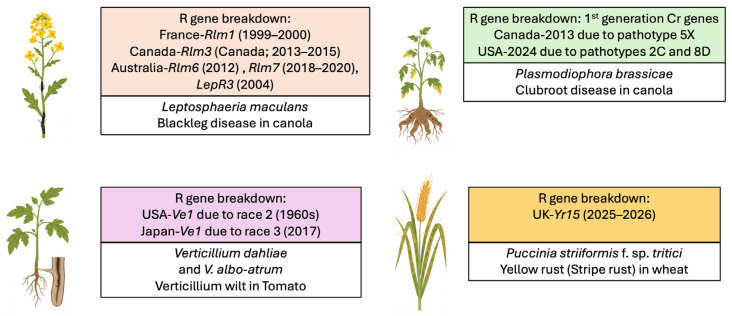
Resistance gene breakdown in major crops. The figure illustrates that R genes have been overcome by evolving pathogen races or pathotypes across different geographic regions.

Researchers generally categorize host immunity into two frameworks, namely major gene resistance and minor gene resistance, based on their genetic complexity and inheritance patterns [19,20]. Major gene resistance mainly depends on the recognition of pathogen effector and plant receptors. The common major gene is the nucleotide-binding leucine-rich repeat (NLR) receptor, which acts as an immune receptor to recognize pathogen effector proteins in host cells during infection [20,21]. The pathogen effectors encoded by pathogenicity-associated genes or virulence factor genes facilitate the infection. Their evolution is driven by the need to bypass plant resistance. If the plant resistance (*R*-gene product) detects the specific pathogen effector, the recognition triggers the HR response to starve the pathogen [22]. Therefore, these pathogen-derived genes are called avirulence (Avr) genes when recognized by the corresponding *R*-gene product [23]. The resistance is often non-durable, race-specific, and associated with an ETI response. The interaction is also defined by the gene-for-gene hypothesis [24]. In contrast to *R* genes, minor genes (pattern recognition receptor genes/PRRs) are surface-level receptors that detect initial microbial attachment [25]. They provide a constant level of surveillance and maintain the signaling machinery that major genes need to function effectively. The resistance is durable, non-race specific, and has a PTI response. Minor genes are highly sensitive to the environment. Because their individual effects are small, changes in soil quality or weather can easily alter the signal of a single minor gene [26]. Quantitative trait loci (QTL) mapping helps researchers account for this by using large populations to identify genetic regions that consistently perform well across different environments [27]. Also, due to repeated use of the same resistance genes and rapid evolution of avirulence genes, there is a continuous cycle of pathogen adaptation and major gene resistance breakdown in plants [28,29] (Figure 1).

For example, *R* genes in canola (*Brassica napus* L.)—*Rlm1*, *Rlm3*, *Rlm6*, *Rlm7*, *LepR1*, and *LepR3*—showed the resistance breakdown against pathogen *Leptosphaeria maculans*, which causes blackleg disease in France, Canada, and Australia [29,30,31,32]. The clubroot disease, caused by *Plasmodiophora brassicae*, poses a significant threat to canola production in Canada [33]. The first generation of resistance to clubroot provides good protection in most canola fields [34]. However, a recent survey revealed a breakdown of first-generation resistance to clubroot in three resistant canola cultivars in North Dakota [35]. In addition, there has been a gradual decline in resistance for varieties carrying only a single CR gene, particularly those with Crr [36]. In tomato (*Solanum lycopersicum*), *Ve1* and *Ve2* encode receptor-like proteins to recognize Ave1 and Ave2 effector proteins of *Verticillium dahliae* and provide race-specific resistance to Verticillium wilt [37]. With the emergence of new races like *V*. *dahliae* race 3, this resistance variety *Ve1*, containing tomato cultivars, showed the disease development [38,39]. In wheat diseases, yellow rust (Stripe rust) caused by the pathogen *Puccinia striiformis* f. sp. *tritici* affects wheat throughout the world [40]. *Yr15* is a major gene that has been widely used but has recently been overcome by new yellow rust strains in Europe [41,42]. To extend the major gene breakdown, another devastating fungus, *Magnaporthe oryzae* (also known as *Pyricularia oryzae*), causes rice blast disease in Asia [43]. The management is mainly controlled by host resistance genes and by understanding the race structure of the pathogen [44]. Though these strategies are constantly challenged by new pathogen races, diverse durable resistance sources are needed to control the adapted pathogen races [45,46].

## 3. The New Frontier for Durable Resistance

Susceptibility genes are plant genes that facilitate the establishment of a compatible interaction with a pathogen [16]. Unlike R genes, which actively detect and fight invaders, S genes are exploited by pathogens for host recognition, immune suppression, and nutrient acquisition [47]. R genes are usually race-specific and can be easily broken by evolving pathogens. Though the S genes are essential for the pathogen’s basic infection strategy, mutating them creates a hurdle that is much harder for the pathogen to overcome. Many S genes are used by a wide range of pathogens [17,18].

Many plant pathogen effectors, proteins that interfere with host proteins and cellular processes, facilitate susceptibility and disease development. Whether identified as natural variants or through targeted mutagenesis, recessive gene alleles serve as a source of plant resistance [18,48]. For example, a mutation in the mildew resistance locus O (*mlo*) gene in barley [49], which encodes a membrane protein that is needed for the negative regulation of immunity, provides resistance to almost all races of powdery mildew pathogens across different plant species [50]. A natural mutation discovered in Ethiopian barley germplasm has served as the foundation for *mlo*-mediated resistance, a strategy that has provided durable protection in European agriculture for almost four decades [51]. Interestingly, *MLO* silencing in cucumber confers resistance to the fruit rot fungus, *Corynespora cassiicola* [52]. The *EDR1* gene serves as a negative regulator of plant immunity, and its protein kinase activity inhibits the association of immune regulators PAD4 and EDS1. By inducing a mutation in *EDR1*, the plant can form the EDS1-PAD4 complex necessary for an active immune response [53]. While this mechanism was detailed in *Arabidopsis*, its practical utility has been demonstrated in complex crops like hexaploid wheat, where CRISPR-based editing of the three *EDR1* homeologs confers significant resistance to powdery mildew [54]. Moreover, TaPsIPK1 phosphorylates the transcription factor TaCBF1d, which in turn reduces the expression of defense-related genes and increases the expression of S genes. Inactivation of TaPsIPK1 by genome editing confers broad-spectrum resistance against rust without a yield penalty [55,56]. Plant immunity can be undermined by specific metabolic processes. In wheat, resistance to rust has been linked to the varying expression of genes involved in amino acid metabolism. Specifically, when the branched-chain aminotransferase 1 gene (*TaBCAT1*) is disrupted, wheat exhibits a decrease in susceptibility to both stem and yellow rust [57]. The PvRXLR111 effector from *Plasmopara viticola* stabilizes the VvWRKY40 protein in grapes during infection [58]. The significance of this interaction is highlighted by studies in the model plant *Nicotiana benthamiana*, where silencing the orthologous gene led to a noticeable decrease in susceptibility to the oomycete *Phytophthora capsica* [58]. Common symbiosis genes appear to function as S genes in various plant-pathogen interactions [59]. This was discovered by examining the structural similarities between beneficial mycorrhizal haustoria and the invaginations created by pathogens [60]. Experimental data show that mutations in essential genes for symbiosis, specifically *API* and *RAD1* in legumes and their orthologs in *Arabidopsis*, confer resistance against oomycete pathogens by hindering their ability to exploit the host [61,62]. In potato, the gene *NRL1* (an NPH3/RPT2-LIKE1 protein) normally acts as a component of the CULLIN3 ubiquitin E3 ligase complex to facilitate infection by the oomycete *P. infestans* [63]. When *NRL1* is silenced, the immune-promoting protein SWAP70 is stabilized rather than degraded, thereby enhancing resistance. This gene, along with the MAP3K-encoding StVIK, represents a growing list of susceptibility factors identified through the study of late blight effectors [64].

Bacterial TALs enter the plant cell nucleus, bind to specific promoter sequences, and hijack the plant transcription machinery to support a successful infection [65]. Recent research highlights that identifying S genes through pathogen effectors and their corresponding host range would be helpful [66]. The SWEET sugar transporters act as a “susceptibility hub” because they are exploited by a wide variety of pathogens [67]. While originally the transcriptional induction of sugar transporters was linked to TAL effectors, pathogens that lack TALs also induce the SWEET sugar transporters, especially root-knot nematodes [68], clubroot *Plasmodiophora brassicae* [69], and *Pseudomonas syringae* [70]. Targeting TAL binding sites in the promoter regions of three major *SWEET* genes in rice has demonstrated broad-spectrum resistance to *Xanthomonas oryzae* pv. *oryzae* [71]. As in barley, *MLO* silencing in pepper also confers resistance to the bacterium *Ralstonia solanacearum* [72]. The dominant rice *Xa13* gene encodes a *SWEET11* sugar transporter that is hijacked by the *Xanthomonas* effector PthXo1 to provide nutrients for bacterial growth [73]. In contrast, the resistant allele contains promoter mutations that prevent this effector from binding, thereby cutting off the pathogen’s nutrient supply and suppressing infection [74]. The *DMR6* (*DOWNY MILDEW RESISTANT 6*) gene functions as a susceptibility factor against various biotrophic pathogens by regulating plant hormone levels [75]. Specifically, it encodes an oxygenase that reduces the plant immune response by hydroxylating salicylic acid (SA) [76]. Through genome editing, researchers have successfully developed tomato plants with higher resistance to *Xanthomonas* and potato varieties with reduced susceptibility to late blight (*Phytophthora infestans*) [77]. Furthermore, in rice, the induction of the *DMR6* ortholog by *Xoo* and *Xoc* TALes indicates that pathogens can deliberately trigger these host negative regulators to facilitate infection [78]. The fire blight bacterium *Erwinia amylovora* infects plants through the DspA/E effector, which interacts with the apple susceptibility protein MdDIPM4 [79]. The *MdDIPM4* knockout has been produced in two *Malus domestica* susceptible cultivars using the CRISPR/Cas9 system and has demonstrated a significant reduction in susceptibility to fire blight disease [80].

S genes are required at all the stages of viruses, including initial disassembly, genome replication, RNA translation, and systemic movement [81]. The interaction between the p23 protein of the beet black scorch virus (BBSV) and heat shock protein (Hsc70-2) is essential to organize the replication in the endoplasmic reticulum [82]. The overexpression of Hsc70-2 increases the BBSV accumulation, and silencing the protein prevents the formation of replication complexes entirely [82]. For cell-to-cell movement, viruses need the ER of the host plant, and the Tomato spotted wilt virus protein is associated with the ER [83]. The mutated protein of RNA-dependent RNA polymerase 3 (*rdr3*) in *Arabidopsis thaliana* delays the cell-to-cell movement [83]. Infections by begomoviruses can trigger the host to produce rgs-CaM (a calmodulin-like protein), which functions as a negative regulator of the plant defense system [84,85]. By suppressing RDR6 transcription and targeting SGS3 for degradation, rgs-CaM effectively prevents the amplification of gene silencing. This disruption of the host’s RNAi machinery allows the virus to accumulate more successfully within the plant [84].

The diverse examples of S genes across fungal, bacterial, and viral pathosystems, ranging from sugar transporters like *SWEET* to immune regulators like *DMR6*, demonstrate that susceptibility is not a single gene event but a complex network of hijacked host processes. Identifying these hidden targets within the massive genomes of a crop and discovering how R genes break down remains a significant challenge that traditional phenotypic screening alone cannot resolve. The integration of multi-OMICS technologies has revolutionized this discovery phase by providing a systems-level view of the infection process. By integrating transcriptomic analysis of effector-triggered host responses with metabolic profiles of nutrient diversion and genomic mapping of mutant populations, researchers can now move beyond the unforeseen discovery of the vulnerable genetic centers in the host machinery.

## 4. Discovery of Long-Term Resistance in Plants

Omics platforms have reshaped our understanding of plant-pathogen interactions by decoding the complex molecular signals of resistance and susceptibility. Through the study of genomics, transcriptomics, proteomics, and metabolomics, scientists can now map the entire progression of disease in detail. By identifying the underlying molecular networks and resistance factors, these approaches provide a roadmap for modern crop breeding and agricultural disease control. This progress is improved by artificial intelligence (AI) and high-throughput sequencing, which together enable the rapid interpretation of complex biological data. These advancements support the development of sophisticated models to predict how genes, proteins, and metabolites interact during a pathogen attack.

### 4.1. Genomics

Following the landmark publication of the initial *Arabidopsis* and human genome drafts in the early 2000s, the field of genomics has seen an extraordinary period of growth over the subsequent quarter-century [86]. The researchers were able to understand the function of genes, mutations, and phenotypic changes through sequencing and annotating genomes [87]. Also, the use of comparative genomics to study both hosts and pathogens has helped to understand important genes responsible for immunity, resistance, and virulence, effectively decoding the molecular foundations of defense and susceptibility [88]. Specifically, whole-genome sequencing of pathogens has enabled comprehensive sequencing of numerous high-profile plant pathogens, providing a molecular blueprint for disease management and resistance breeding. Significant milestones include sequencing of *X. oryzae* (bacterial blight) [89], the oomycete *P. infestans* (late blight) [90], and the wheat pathogen *Blumeria graminis* (powdery mildew) [91]. Other significant genomic assemblies include *P. syringae* (bacterial canker) [92], *X. axonopodis* (bacterial blight) [93], and the economically devastating *Fusarium graminearum* (Fusarium head blight) [94]. Furthermore, the sequencing of *Puccinia triticina* (leaf rust) [95], *Ralstonia solanacearum* (bacterial wilt) [96], *Alternaria solani* (early blight) [97], and *L. maculans* [98] has enabled researchers to identify specific virulence factors and host-susceptibility targets. By decoding these genomes, scientists can better understand the infection mechanisms, such as hormone modulation and nutrient hijacking, that pathogens use to overcome plant defenses. From the host perspective, genomic advancements have facilitated the discovery of both R genes and S genes, which are the primary regulators of the plant immune system.

The *Arabidopsis thaliana* genome has been an essential model for mapping the NLR gene family, a group of receptors critical for detecting pathogens [99]. In a practical application, rice genomics has enabled precision breeding to “stack” various resistance genes such as *Xa21* and *Pi9*, resulting in varieties with robust, broad-spectrum protection against fungal and bacterial threats [99]. Advancing into pan-genomics, researchers can study the entire genetic diversity within a specific species, relying on multiple cultivars, including wild relatives, and acknowledging that one individual cannot possibly contain all the genes present in a diverse population [100]. In recent breakthroughs in wheat research, over 2000 global wheat accessions and more than 47,000 phenotyping records spanning multiple environments and various pathogen races were analyzed to identify broad resistance to yellow rust (YR) [101]. The study identified a massive library of 431 YR resistance loci that contribute to both all-stage and adult-plant resistance. Beyond mapping, the study successfully cloned three critical resistance genes that provide protection against both YR and powdery mildew. One superior allele, TaEDR2-B, was found to provide broad-spectrum resistance without a yield penalty [101]. In another study, genetic mapping indicated that the protection governed by the single gene Yr84, exhibiting incomplete dominance, and using bulked segregant analysis sequencing (BSA-Seq), the team localized the resistance region on the chromosome and provided information for KASP (kompetitive allele-specific PCR) marker development in marker-assisted selection [102]. In addition to pathogenic genomic information, identifying the whole genome sequence of the host plant also provides information to develop disease resistance gene-cloned varieties [103] and susceptibility gene mutated varieties using gene editing technology.

Although S-gene knockouts have yet to be developed in canola, the persistent failure of traditional R-gene strategies has redirected breeding efforts toward susceptibility factors. The S-gene inactivation via CRISPR/Cas9 or Targeting Induced Local Lesions in Genomes (TILLING) offers a promising pathway for achieving durable, broad-spectrum resistance against virulent pathotypes [104,105,106]. Susceptibility factors in canola primarily function by modulating host physiology and are generally categorized into three functional domains: hormonal manipulation [107], nutrient allocation [69], and immune suppression [108]. These host proteins act as negative regulators of plant immune signaling, and knocking out these genes can confer resistance in the plant. Comparative genomics enables new findings by allowing researchers to identify critical genetic differences between species and strains, which is essential for understanding pathogen evolution and host resistance [109]. In addition, it reveals the key adaptive mechanisms in pathogen host-niche specialization and pathogen dynamics, where pathogens evolve effectors to suppress plant immunity, and plants evolve R genes to detect them [110,111,112,113]. Utilizing genome-wide association studies (GWASs) in pathogen interaction studies facilitates the identification of genomic regions or variants associated with increased susceptibility across diverse populations [114]. A recent GWAS of *A. thaliana* identified *RDR6* and *DCL2* as negative regulators of resistance to cucumber mosaic virus [115]; a GWAS on *Gossypium hirsutum* (upland cotton) identified that specific Toll/interleukin 1 (TIR) nucleotide-binding leucine-rich repeat receptors act as negative regulators. A specific cluster of these receptors (TIRP1) was found to be involved in self-association and auto-activity, which surprisingly results in compromised resistance to *Verticillium dahlia* [116]. Studies in *Arabidopsis* identified components of cytokinin signaling as negative regulators of immunity during *R. solanacearum* infection, particularly in inhibiting root growth as part of the defense-pathogen interaction [117]. The identification of host factors that negatively regulate defense provides a library of potential S-gene targets for crop improvement.

### 4.2. Transcriptomics

Transcriptomics represents the study of the complete set of RNA transcripts, including mRNA and various small RNAs, which allows researchers to understand the genetic activity at a specific time [118]. Initial frameworks include microarrays, expressed sequence tags, and serial analysis of gene expression, and then RNA sequencing (RNA-seq), single-cell RNA-seq, and special transcriptomics have emerged as novel approaches to their technical precision and broad applications [119,120]. The first adopted transcriptional profiling was used to investigate the interaction of *Phytophthora infestans* [121,122]. Since then, a diverse group of pathosystems has been decoded, contributing insights into the genetic pathways leading to pathogen virulence and host immunity [123,124,125,126,127,128].

Through the application of RNA sequencing, researchers can construct comprehensive gene expression maps within specific tissues [129]. These profiles clarify the activation of pathogen-recognition receptors and the intricate signaling networks controlled by defense-related phytohormones, including salicylic acid, jasmonic acid, and ethylene [130]. Furthermore, transcriptomic datasets offer insight into how plants regulate physiological defenses, such as the strengthening of cell walls, the generation of reactive oxygen species (ROS), and the induction of programmed cell death [131]. Beyond host responses, these studies are instrumental in identifying how pathogen-secreted effectors hijack host genetic machinery to dampen immunity or enhance infection [132]. Analyzing the simultaneous transcriptomes of both the host and the pathogen during their interaction provides a complete view of the molecular patterns and signaling events that define the infection process [133]. Once these candidate genes undergo functional validation, they become high-value targets for precision breeding strategies designed to bolster durable disease resistance in crops [134].

Susceptibility gene interactions in host-pathogen recognition often result in upregulation of genes involved in cell wall modification, nutrient transport, and immune response suppression. RNA-seq identified resistant and susceptible genes in tobacco cultivars in response to infection by *P. nicotianae*, where upregulated genes are directly related to susceptibility [135]. Comparative transcriptome analysis revealed potential susceptibility factors associated with wheat stem rust (*P. graminis* f. sp. *tritici*), and these genes are linked to cell death suppression and photosynthesis impairment [136]. RNA-seq has been used to uncover several classes of negative regulators, particularly in model plants like *Arabidopsis* and various crops. *AtRTP5* (Arabidopsis thaliana Regulators of Trafficking Protein 5) was identified via RNA-seq and gene expression analysis as a negative regulator of immunity to *Phytophthora* pathogens, acting by modulating salicylic acid (SA) and jasmonic acid (JA) levels [137]. *FvNPRL-1* in strawberry has been identified through RNA-seq and characterization as a negative regulator of defense against anthracnose [138], and *CML46* and *CML47* have been identified as negative regulators of PTI (PAMP-triggered immunity) against *P. syringae* [139]. These negative regulators are potential susceptibility genes and are crucial for agricultural biotechnology, as targeting these negative regulators through methods like CRISPR/Cas9 editing can create resistant, high-yielding crops.

While transcriptome profiling is effective, it often identifies many candidate genes that require functional validation, such as through gene editing tools, to confirm their role in susceptibility. Additionally, integrating transcriptomics with other omics data, such as metabolomics and proteomics, can provide a more comprehensive understanding of the infection process.

### 4.3. Proteomics

Proteomics involves the large-scale identification and characterization of the total protein profile within a specific tissue, as well as the structural determination of proteins. This field originated in 1975, when researchers first employed two-dimensional gel electrophoresis (2-DE) to map the proteome of *Escherichia coli* [140]. In plant science research, proteomic tools are essential for understanding how various proteins control growth and development and for uncovering how the proteome shifts in response to biotic stressors such as infection. For example, plant fluid proteomics investigates the specialized protein compositions of the apoplast, xylem, and phloem to map the critical molecular changes where hosts and pathogens interact [141]. By analyzing these distinct compartments, researchers can identify early defense signals in the apoplast, effector movement within the xylem, and the systemic signaling mechanisms that regulate whole-plant immunity [142].

By investigating diverse plant pathosystems, proteomics has successfully identified critical proteins involved in the host-pathogen interaction [143,144]. These studies highlight the molecular drivers of host susceptibility and resistance, providing a foundation for designing more sophisticated and effective disease management protocols [145]. Building on these early advancements, several researchers have utilized the 2-DE method to discover the proteomes of plant pathogens, successfully identifying proteins that drive both pathogen virulence and host defense mechanisms [146,147,148,149]. Specific protein spots that show differential expression (e.g., increased in susceptibility) are excised from the gel and identified using mass spectrometry (MS) or MS/MS [150].

Beyond the fields of genomics and transcriptomics, mass spectroscopy is important for high-throughput profiling because of its remarkable sensitivity, high-resolution mass separation, rapid data acquisition, and the ability to derive structural details through molecule fragmentation [151]. MS has been instrumental in uncovering the complex regulatory mechanisms of RIN4, including its phosphorylation and cleavage by pathogen effector proteases like AvrRpt2 [152], and has identified a novel RNA-binding protein that suppresses plant immunity against *Phytophthora capsici* [153]. MS-based proteomics (e.g., ubiquitylomics) has revealed that E3 ubiquitin ligases and related enzymes often act as negative regulators by targeting positive immune regulators for degradation [154]. Moreover, MS has identified a family of Ca^2+^-dependent membrane lipid-binding proteins that act as negative regulators of disease resistance against *P. syringae* and *Hyaloperonospora parasitica* [155]. These specific attributes allow MS to effectively process intricate biological samples that exhibit a wide range of molecular concentrations [156]. In proteomics, peptide identification relies on peptide-spectrum matching (PSM). This process involves using specialized search engines to compare experimental MS2 spectra, which are fragments of the original MS1 precursor ions, against a database of predicted fragment ions derived from the organism’s known protein sequences [157]. Modern proteomic analysis is increasingly enhanced by deep-learning models that improve matching accuracy by integrating variables such as ion mobility and retention time alongside traditional metrics like mass-to-charge ratios and fragmentation signatures [158,159,160]. By allowing for the coexisting analysis of thousands of proteins, this approach offers deep insights into the molecular interaction between plants and their pathogens, effectively revealing the complex mechanisms that drive host immunity and pathogen infectivity [161].

AlphaFold, the AI system developed by Google DeepMind, has fundamentally changed how we approach proteomics [162]. Predicting the 3D structures of proteins with high accuracy has enabled researchers to identify theoretical models based on known homologous structures. These models facilitate the structural simulation of host-pathogen interfaces, allowing the modeling of predicted host proteins with experimentally identified pathogen effectors to uncover potential susceptibility proteins.

### 4.4. Metabolomics

Metabolomics involves the analysis of small molecules to provide comprehensive insights into various aspects of plant biology, including growth patterns, developmental stages, and responses to external stressors. Furthermore, it serves as a critical tool for identifying phenotypic diversity resulting from either environmental shifts or genetic differences [163]. Unlike other omics fields, metabolomics is often viewed as a more accurate reflection of the molecular phenotype, as the concentration of metabolites represents the final integration of various upstream biological activities [164,165].

Methodologically, metabolomic research is divided into targeted and non-targeted strategies [166]. The primary technological platforms include nuclear magnetic resonance (NMR) and mass spectrometry (MS) coupled with gas or liquid chromatography. Using these techniques in tandem often yields complementary data, significantly expanding the detectable metabolic profile. While the influence of specific metabolites on plant disease has been documented for many years [167], the first true omics-scale metabolic profiling of a host-pathogen system was conducted by Allwood et al. (2006) [168]. Their work identified phospholipids, specifically phosphatidic acid and phosphatidylglycerol, as the primary non-polar markers distinguishing the *Brachypodium distachyon* response to *Magnaporthe grisea*. Subsequent research, such as the study by Doehlemann et al. (2008) [169], integrated transcriptomics with metabolomics to map the changes in maize during infection by the biotrophic fungus *Ustilago maydis*.

The growing use of metabolomics in plant pathology not only clarifies biochemical defense mechanisms but also aids in the discovery of biomarkers for disease resistance [170]. The biomarkers are specific metabolic signatures, such as pathogen-related metabolites that serve as indicators of a plant defensive state [171]. These biomarkers, which may include phenolic compounds, alkaloids, or specific amino acids, are also used in metabolic profiling of large populations [172]. By identifying these chemical fingerprints early in the plant developmental stage, researchers can utilize them as selection criteria to predict the resistance level of germplasm without waiting for full disease symptom expression or high-cost field trials [173]. This information is vital for creating modern crop protection strategies and enhancing agricultural durability. The studies and techniques summarized here support the identification of the specific genes, proteins, and metabolites that drive plant immunity or allow pathogens to exploit their hosts.

Through the study of genomics, transcriptomics, proteomics, and metabolomics, scientists can now map the entire progression of disease in detail [174]. By identifying the underlying molecular networks and resistance factors, these approaches provide a roadmap for modern crop breeding and agricultural disease control. For example, key genes related to bacterial leaf streak were identified using genomics and RNA-seq analyses [175], and the crystal structure of Avr proteins was experimentally determined using multi-omics approaches, such as the AvrM effector protein of flax rust [176] and different Avr effectors of powdery mildew [177]. Moreover, a multi-omics approach was used to identify the binding counterparts of the MLO protein [178]. Knockout of MLO genes, confirmed by MS and gene expression studies, leads to reduced susceptibility without negatively impacting plant quality [52]. To identify the susceptibility responses, such as cell wall modifications and the modulation of nutrient and phytohormone balance, a multi-omics approach was utilized [179,180,181]. The progress is accelerated by artificial intelligence (AI) and high-throughput sequencing, which together enable the rapid interpretation of complex biological data. These advancements support the development of sophisticated models to predict how genes, proteins, and metabolites interact during a pathogen attack.

## 5. Managing Pleiotropy and Fitness Cost

A major challenge in achieving durable resistance is the inherent trade-offs between plant responses to pathogens [182]. This fitness cost is driven by mutual interference between signaling processes such as the salicylic acid (SA)-mediated pathway, which targets biotrophs, and the jasmonic acid (JA)-mediated pathway, which protects plants against necrotrophs [183]. These signaling networks do not operate separately; they interact with growth-regulating DELLA proteins and other hormonal pathways to balance defense with plant development [184]. In cereal crops, one of the specific gain-of-function *Rht* alleles (GOF: genetic mutations that confer a novel or enhanced activity of a protein rather than deactivating a gene), *Rht* (reduced height), often forms gain-of-function forms of DELLA proteins that are insensitive to GA [185]. The wheat plants carrying multiple *Rht* GOF alleles often exhibit extreme dwarfism and increased vulnerability to biotrophic pathogen, *B. graminis*. However, these same alleles offer significant defensive response against necrotrophic and hemibiotrophic pathogens such as *Oculimacula spp*. and *F. graminearum* [186]. This highlights how manipulating plant height through GOF alleles can fundamentally shift the balance between SA- and JA-mediated defenses. As discussed in the previous section, the use of *mlo* in barley breeding programs serves as a primary example of how manipulating susceptibility can have different impacts on different pathogens. While mutant *mlo* alleles have been widely deployed to secure durable protection against powdery mildew with some pleiotropic effects [49], they often carry a metabolic burden that results in a grain yield loss of approximately 5–15% [187]. This pleiotropic effect is characterized by spontaneous leaf cell death, which reduces the overall photosynthetic capacity and productivity. Also, increases susceptibility to *Ramularia* leaf spot disease and wheat blast fungus [188]. However, plants and breeders have found ways to overcome or minimize this, including the utilization of specific mutant alleles, genetic background optimization, and refined genetic engineering [189,190]. Another example, *dmr6* mutants, display growth reduction due to higher levels of endogenous SA accumulation in the plant [191]. However, *dmr6* mutants have shown enhanced resistance to *Phytophthora* (late blight), *Xanthomonas* (bacterial spot), and *Pseudomonas* [77,192]. Also, CRISPR-edited *dmr6* mutants with increased tolerance to late blight showed drought tolerance in tomato [193]. While knockdown of GAPDH confers resistance to bushy stunt virus infection, its knockdown leads to enhanced susceptibility to tobacco mosaic virus and bamboo mosaic virus [194,195]. Similarly, knockdown of translational initiation factor eIF4E1 provides protection against clover yellow vein virus, but causes hypersensitivity to turnip mosaic virus [196]. Promisingly, S-gene manipulation does not always result in unfavorable trade-offs. A significant number of examples show that S-gene mutants achieved robust resistance without any detectable decline in agronomic performance, such as *SWEET* poly mutants in rice for bacterial blight [71], *TaEDR1* for powdery mildew [54] *TaMLO1* for powdery mildew [197], *TaMKP1* for yellow rust and powdery mildew [198], *TaCIPK14* and *TaPsIPK1* for stripe rust [56,199], *TaGW2* for leaf rust [200], *TaIMP-α* for barley yellow dwarf virus [201], and *TaPDIL5-1* and *TaeIF4E* for wheat yellow mosaic virus [202].

Compared to R genes, knocking out S genes in plants provides durable resistance against pathogens because it does not depend on the specific recognition of pathogen effectors, which pathogens can easily evolve to evade [203]. However, to mitigate the evolutionary risks associated with S-gene breakdown, researchers must adopt a combined approach to protect crops. Integrating S-gene mutants with minor gene resistance or targeting conserved regions of pathogen proteins can create a more resilient defensive barrier. Furthermore, precision tools like CRISPR enable the simultaneous modification of multiple homologs, preventing genetic compensation and S-gene-driven undesirable trade-offs. To prevent loss of plant vigor, breeders can utilize pathogen-inducible promoters that ensure S-gene silencing occurs only during an attack or substitute growth-compromising alleles with functional homologs that maintain physiological growth while minimizing susceptibility [204,205].

## 6. Conclusions and Future Remarks

Plant protection is shifting from resistance genes to a complete understanding of host-pathogen molecular interactions. As outlined in this review, the traditional reliance on major R genes often leads to rapid resistance breakdown due to the high evolutionary plasticity of pathogen effectors [206]. The integration of multi-omics approaches, including genomics, transcriptomics, proteomics, and metabolomics, has revealed that durable immunity is not the result of a single genetic switch but rather a complex interplay between surface-level PTI surveillance and the strong intracellular ETI response.

Perhaps the most significant revelation of recent years is the role of susceptibility genes. By identifying host genes, such as the *MLO* locus, *SWEET* transporters, and *DMR6*, researchers have developed broad-spectrum resistance that is much harder for pathogens to overcome. Multi-omics platforms serve as tools to identify how these interactions are decoded, allowing researchers to move beyond trial-and-error breeding toward precision genome engineering. While progress in multi-omics integration is extensive, several frontiers remain to be explored to complete the story of host-pathogen interaction. Most current omics data are bulk, meaning they average the signals of an entire tissue. However, infection happens at the single-cell level. Future directions must include spatial transcriptomics to determine which genes are hijacked in the specific cells surrounding a fungal haustorium or a bacterial colony. The integration of AlphaFold represents a new area of study. Future research should focus on high-throughput interactome mapping using AI to predict the binding affinity between thousands of pathogen effectors and host proteins before the pathogen even reaches a new geographical region. This predictive breeding could allow researchers to engineer resistance to emerging races proactively. Finally, moving metabolomics from the lab to the field via portable nanosensors could enable real-time detection of susceptibility biomarkers. This would enable farmers to apply targeted treatments before visible symptoms appear, slowing the cycle of pathogen adaptation. The transition from finding a gene to understanding a network is the main point of the multi-omics era. By disabling the host machinery that pathogens require S genes while strengthening the plant’s internal surveillance genes, we can finally break the “boom-and-bust” cycle and ensure sustainable global crop production.

## Data Availability

No new data were created or analyzed in this study. Data sharing is not applicable to this article.
